# Exposome-wide Association Study for Metabolic Syndrome

**DOI:** 10.3389/fgene.2021.783930

**Published:** 2021-12-07

**Authors:** Peng Gao, Michael Snyder

**Affiliations:** Department of Genetics, Stanford University School of Medicine, Stanford, CA, United States

**Keywords:** type 2 dabetes, exposome-wide association study, pathophysiology, etiology, multi-omics analyses, exposomics, metabolic sydrome

## Introduction

Metabolic syndrome (MetS) is a group of conditions that happen concurrently which increases the risks of stroke, cardiovascular diseases, and type 2 diabetes. These conditions include elevated blood pressure, excessive waist fat, high blood sugar, and abnormal triglyceride or cholesterol levels. The increasing rate of MetS is becoming a health problem worldwide (Rochlani et al., 2017). MetS and its components are caused by the complex interactions between various genetic, behavioral, and environmental factors, whose detailed mechanistic understanding is unclear. Previously, significant advances have been made in identifying the genetic determinants, epigenetic alterations, and proteomic/metabolic networks that play essential roles in the etiology and progression of MetS (Dizaji, 2018). However, we are still uncertain about what environmental and lifestyle triggers the onset and progression of the individual’s abnormal metabolic alterations. As such, the genetic heritability of the MetS only ranges from 10 to 30%, indicating both genetic and environmental factors play essential roles (Musani et al., 2017). Although several contributing factors have been raised, such as insulin resistance, chronic inflammation, adipose tissue dysfunction, oxidative stress, microbiome imbalance, and circadian rhythm disorders, it is challenging to clarify which of them are triggers or results of MetS (Xu et al., 2018).

The exposome concept was first introduced in 2005, which aims to investigate the relationships between the external exposures and internal -omes in the progression and occurrence of the disease, which can be an essential complement to the conventional etiology studies which primarily have focused on genetics (Wild, 2005). The comprehensive exposome refers to the total exposure in all life courses from conception onwards, and exposomics investigates external and internal exposures and how they collectively determine health and disease risks (Vermeulen et al., 2020). Practically, a detailed protocol that merges exposure monitoring devices and experimental pipelines to determine abiotic and biotic external exposome on the personal level has already been implemented (Jiang et al., 2018, Jiang et al., 2021). The exposomics research includes the studies of the external and internal exposome and investigating how they holistically impact human health and disease risks. Exposome-wide association study (EWAS) provides novel insight for investigating the roles and relationships of diverse chemical, biological, and physical exposures in the entire life process, the key developmental stages of the disease, and the etiology and progression across generations (Li et al., 2019). Specifically, EWAS usually applied advanced analytic methodologies to find the significant associations between phenotypes and the exposome (Patel, 2019). It is a data-driven approach that can fit the application of both population health and individual differences so that the complex relationship between external and internal environments as well as the life-course environmental health can be systematically explored (Chung et al., 2019). Overall, this article aims to outline the feasibility of leveraging the interdisciplinary EWAS to understand MetS in more depth, focusing on the interactions between different types of environmental exposures and internal physiological factors, and underlying mechanisms in the pathogenesis of MetS and its components such as cardiovascular disease (Juarez et al., 2020).

## Chemical Exposures and MetS

Chemical exposures through different pathways have been found to have pervasive interactions with human metabolites, and increasing evidence is demonstrating that exposures to certain chemicals in our environment play vital roles in the pathophysiology and etiology of metabolic diseases. Also, exposures that occur in early life may have a more profound impact on the lifetime risk of MetS. For instance, exposures to a range of environmental contaminants can contribute to the development of MetS, such as various agrochemicals, pharmaceuticals and personal care products, food toxins, flame retardants, and heavy metals, and certain of them present endocrine-disrupting characters (De Long and Holloway, 2017; Silva et al., 2020). Endocrine-disrupting chemicals (EDCs) mimic the structures of hormones, which disrupt the normal functions of human metabolism, including various metabolically active essential organs. In addition, the epigenetic alterations caused by EDCs can permanently change the epigenome in the germline, thereby passing the changes to offspring. The metabolic effect of this multi-disruption is the underpinnings of metabolic diseases, which are clinically manifested as MetS and its components (Papalou et al., 2019). However, since the conventional risk assessment procedure is based on the toxicity of a single stressor or a single group of stressors, it is not suitable for capturing multiple environmental exposures in combination and at low doses (Gao et al., 2018; Gao, 2021). Though the traditional risk assessments assume that each chemical stressor individually acts in a dose-response linear relationship, research is already demonstrating that the combined effect of EDCs is not simply the sum of individual toxicities and does not follow the linear rule (Le Magueresse-Battistoni et al., 2018). Therefore, EWAS studies may have the ability to discover the complex relationships between multiple stressors and identify the key environmental chemical exposures that trigger MetS.

In addition to EDCs, it was found that long-term exposures to particulate matter and air pollutants were positively correlated with the prevalence of MetS in adolescents and children (Zhang et al., 2021a). For instance, an environmental epidemiology study found strong associations between air pollutants and impaired fasting glycemia, and associations between air pollutants and waist-circumference-based obesity, suggesting that chronic exposures to air pollutants may lead to MetS by specifically impacting insulin resistance of the individuals (Eze et al., 2015). This result is consistent with other epidemiology studies, showing the positive associations between ambient air pollution and MetS (Yang et al., 2018; Hou et al., 2020). However, which airborne chemicals contribute to MetS requires further investigation. As such, EWAS studies provide the possibilities to study the associations between the chemical exposures and MetS, generating various testable hypotheses for validating by *in vivo* and *in vitro* experiments.

## Microbial Exposures and MetS

Although the COVID-19 pandemic has dramatically increased the concerns of human exposures to biotic agents in the environment, the links between possible pathogens and MetS have been understudied. Specifically, limited research showed the possible relationships between microbial exposures/infections and MetS. A study showed that pathogen burden presented strong associations with insulin resistance, especially with enterovirus and *Chlamydia pneumoniae* seropositivity, which means that multiple pathogens exposures may lead to low-level and chronic inflammation and then insulin resistance (Fernández-Real et al., 2006). Another study found that long-term exposure to the toxins produced by *Staphylococcus aureus* can cause rabbits to develop glucose intolerance, insulin resistance, and systemic inflammation, which are the symptoms of MetS (Vu et al., 2015). Finally, respiratory viral infections have been associated with type 2 diabetes (Chen et al., 2012) and in at least one case of DNA methylation alterations at metabolic genes were associated with glucose dysregulation (Chen et al., 2018).

In addition to studies of inhalation and dermal exposures to microbes associated with MetS, a larger set of studies have focused on studying the possible ingestion exposures of microbes, gut microbiota, and MetS. Specifically, many studies have examined the potential impact of the gut microbiota on MetS and its relationship with the host metabolism (Dabke et al., 2019) and both animal and human studies have revealed mechanistic links. These relationships include participation in metabolic processes and energy homeostasis, interference with the renin-angiotensin system, and modulation of the host immune system and inflammation. Altering the gut microbiota *via* probiotics, prebiotics, or other dietary interventions may have beneficial effects to mediate MetS (Mazidi et al., 2016). For example, heat-inactivated *Akkermansia muciniphila* helps to relieve the symptoms of MetS in obese and overweight individuals (Dizaji, 2018). In general, the relationships between the biological exposures *via* various exposure pathways and MetS could be further investigated in the future by EWAS studies, along with their interactions with hosts’ microbiome and dietary intake.

## Physical Exposures and MetS

In addition to biotic and abiotic exposome, physical stressors such as noise, heat, and radiation may lead to MetS or exacerbate metabolic diseases. Several epidemiology studies have shown that noise exposures were positively associated with MetS and its components, and increased the risks of MetS (Huang et al., 2020; Li et al., 2021). A similar study also found traffic-related noise and NO_x_ exposures were associated with MetS and its components (Yu et al., 2020). In addition, an *in vivo* study based on the rat model showed that chronic noise exposures may cumulatively alter the occurrence and development of diabetes since those exposures can change the composition of the gut microbiota and induce intestinal inflammation (Cui et al., 2016).

In terms of heat exposure, exposure to exertional heatstroke in female mice led to delayed cardiac metabolic disorders and impact long-term cardiovascular health (Laitano et al., 2020). Moreover, the intracellular expression levels of heat shock proteins may play vital roles in the pathogenesis of MetS (Sabbah et al., 2019). Similarly, another animal model study found insufficient heat stress response may be the basis of susceptibility to MetS (Rogers et al., 2016). Finally, research also found anthropogenic heat emissions were positively correlated with MetS, and this correlation changed with age (Cong et al., 2021).

Various studies have found that high doses of ionizing radiation can cause adverse metabolic and circulatory effects, while low-dose radiation was causally related to circulatory diseases, and the effects were modulated by stress, inflammatory, and immune responses of the individual (Tapio et al., 2021). In addition, radiation therapy may cause MetS. As such, a study showed that MetS could be a sequela of radiation exposure among childhood brain tumor survivors (Cooksey et al., 2019). Similarly, survivors of neuroblastoma and nephroblastoma have been found to have increased risks of developing MetS components, especially for those who had abdominal irradiation (van Waas et al., 2012). Overall, these different studies have demonstrated the possible links between noise, heat, and radiation exposures and the pathogenesis of MetS. However, the related underlying mechanisms require further investigation using additional *in vitro* and *in vivo* experiments.

## Psychosocial Exposures and MetS

Psychosocial assessments can be used in exposure science to decode the exposome triggers of MetS as well. Mental health has been shown to affect MetS (Zhang et al., 2021b). For example, an epidemiology study found the increased prevalence of MetS was significantly associated with higher levels of depression, hostility, anger, and pessimism, and additional adjustment for potential biological mediators had little impact on these associations. Those results can be explained in part by differences in health behaviors and socioeconomic status (Cohen et al., 2010). Similarly, a study found that after adjusting for demographic and lifestyle variables, negative life events and insufficient emotional support may increase the chance of developing MetS. The associations between depression and MetS were found in whites, and anxiety symptoms were significantly related to male MetS. The psychosocial risk index score was positively correlated with the likelihood of suffering from MetS as well (Vogelzangs et al., 2007). Specifically, dysfunctional social networks and experiencing major adverse life events were found to be the potential risk factors for MetS in women, and stress responses (e.g., excessive fatigue and insomnia) may play more significant roles in the development of MetS in men (Pedersen et al., 2016). Furthermore, another study conducted a meta-analysis to determine the association between MetS and psychological stress. The results showed the adults in the low-stress group were 45% less likely to have MetS compared with the adults in the high-stress group with the controls of known covariates. Also, the subsequent meta-analysis based on cross-sectional studies showed that perceived stress had the weakest impact while occupational stress had the strongest impact on MetS (Kuo et al., 2019). In short, significant associations between the psychosocial factors and MetS have been reported, although in all cases the underlying mechanisms are unclear.

## Discussion

In the past decades, various breakthroughs have been made in the identification of internal determinants of MetS which may only play partial roles in the pathophysiology of MetS and its components. Currently, clearly identifying the triggers of personal MetS and accurately predicting personal MetS risk still cannot be achieved. Therefore, determining the environmental factors that are related to MetS, and impact their onset and progression of MetS are highly demanded. Based on the reviewed references in this article, it has been shown that MetS could be caused by the complex interactions between the exposome and the human genome. However, since major current studies are environmental epidemiological studies, the link between these environmental exposures and -omics level studies in relation to MetS remains unclear. Further *in vivo* and *in vitro* studies by leveraging multi-omics approaches (especially metabolomics, lipidomics, and proteomics) are necessary to discover the underlying mechanisms of how environmental factors contribute to MetS. For instance, the application of -omics approaches could help us better understand the individual susceptibility, variability, and risks of MetS, and further advance the research on endocrine disruptors (Messerlian et al., 2017).

Furthermore, utilizing the exposomics approach to reveal unknown environmental factors that cause MetS and EWAS to prioritize all the MetS-related environmental factors would be promising to provide the testable hypotheses for experimental validations (Gao et al., 2021). Nevertheless, the technical barriers in multi-omics and exposomics research do exist, such as the technological challenges on chemical and biological agents identifications (David et al., 2021), the bioinformatics challenges related to the computational throughput and workflows, and the high financial costs of such exposomics cohort study. Fortunately, the results from current large environmental health studies and exposomics related international consortiums including the Global Exposome Harmonization project and others such as the HELIX project provided some insights for future research (Stratakis et al., 2020; Maitre et al., 2021).

**FIGURE 1:**
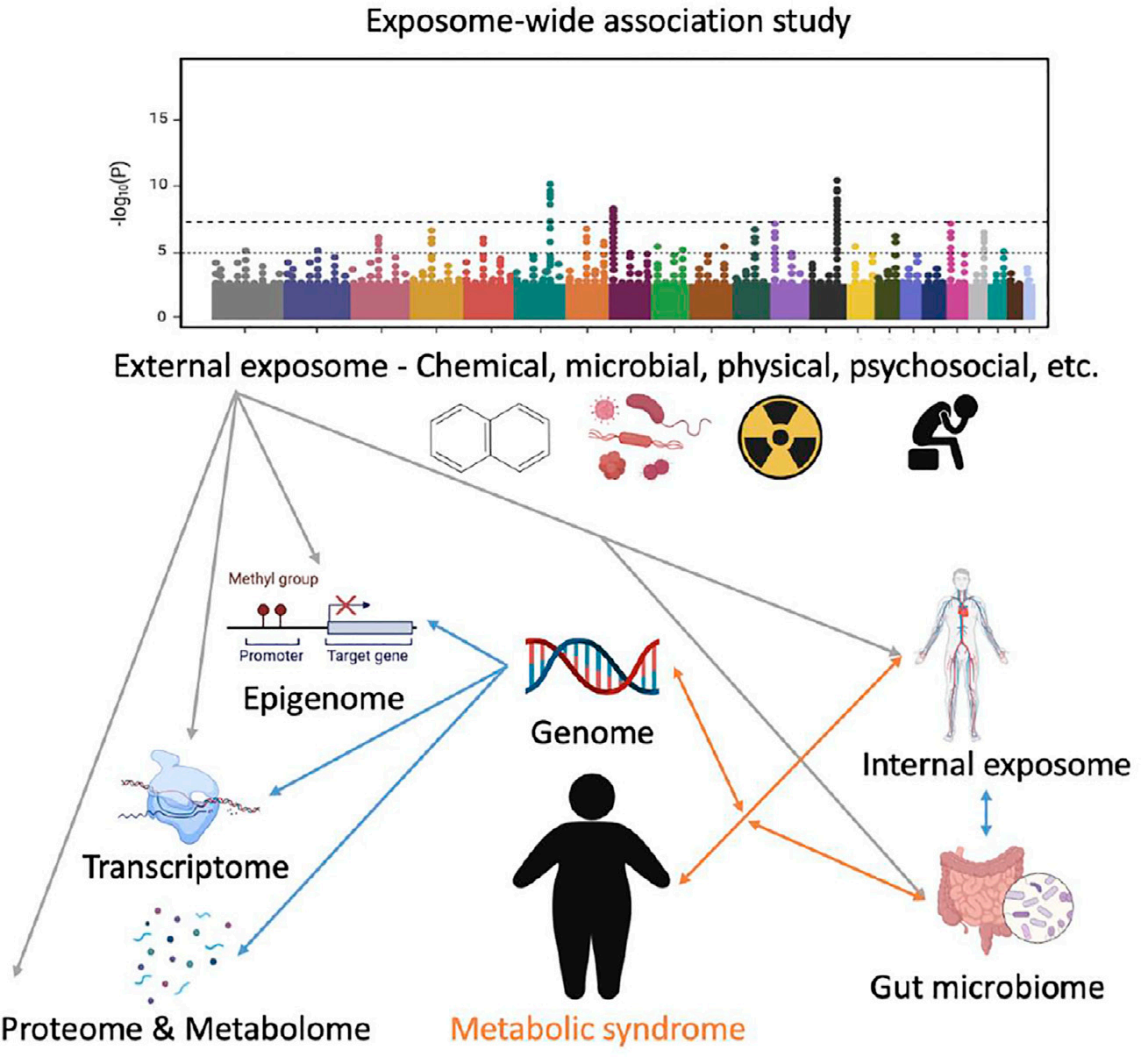
A conceptual framework of applying exposome-wide association study (EWAS) to investigate metabolic syndrome (MetS). The internal exposome includes but is not limited to all the non-endogenous agents inside the human body such as xenobiotics, their biotransformation products, and foreign biological agents. The Manhattan plot was used to illustrate how EWAS could potentially identify the most MetS-related factors in the exposome. Grey lines represent external-internal interactions; blue lines represent intra-internal interactions; and orange lines represent gene–exposome/microbiome interaction. Not all the relationships are presented.

This article aims to provide an overview of the multidisciplinary exposomics studies linked to MetS, with a primary focus on the environmental exposures and their relationships with MetS. Specifically, relationships have been established between the chemical, microbial, physical, and psychosocial exposures and MetS. However, the interactions between them and their holistic impacts on MetS remain unclear. Therefore, generating a framework for utilizing EWAS to investigate the pathophysiology of MetS and its components would be necessary (Figure 1). Novel exposome monitoring approaches and the analytic methodologies of decoding the interactions between the exposome and genome are needed to investigate the interplay between environmental, lifestyle, and genetic factors, as well as the underlying mechanisms in the etiology of MetS. Specifically, the following aspects related to the exposome and MetS need to be further addressed: 1) large cohort research study how the comprehensive exposome is related to MetS; 2) computational and experimental studies of environmental-gene interactions on MetS; 3) big data analytics of integrating the exposome data with internal multi-omics data that are related to MetS; 4) experimental investigation of exposure biomarkers to reflect the MetS-related environmental exposures and predict MetS progression; and 5) development of robust statistical approaches to analyze the impacts of diverse and dynamic exposures on MetS susceptibility (Plusquin et al., 2019). Despite the current challenges, the exposomics and EWAS could be promising and robust approaches to investigate the etiology and progression of MetS and its components in the future due to the relatively low genetic heritability of these diseases.
